# Investigating Genetic Spillage in a *Mimulus cardinalis* (Syn. *Erythranthe cardinalis*) Common Garden Experiment

**DOI:** 10.1002/ece3.74226

**Published:** 2026-08-28

**Authors:** Lydia Duran, Niveditha Ramadoss, Seema Nayan Sheth, Lluvia Flores‐Rentería

**Affiliations:** ^1^ Department of Biology San Diego State University San Diego California USA; ^2^ Department of Plant and Microbial Biology North Carolina State University Raleigh North Carolina USA

**Keywords:** common garden experiments, *Erythranthe*, genetic monitoring, genetic spillage

## Abstract

Common gardens are critical to studying the trait variations in plant species and how environmental or genetic factors influence them. In common gardens, non‐native species and non‐local populations must be monitored closely to reduce their chances of escaping, as this can lead to genetic spillage, which is the introduction of foreign genetic material with potential unwanted phenotypes/genotypes into the native ecosystems. This could decrease the fitness of native populations and threaten natural habitats. This study investigated a possible spillage of genetic material from an existing common garden experiment of the Scarlet Monkeyflower (*Erythranthe cardinalis)*. This garden consists of six populations that originated from three regions across its distribution (North, Central, and South) and were propagated at an Ecological Reserve in Southern California. A population of *E. cardinalis* was recently observed a kilometer away from the common garden, leading to the question of whether it is a wild population or an escapee. Leaf samples were collected from individuals of each provenance established at the common garden, and the “unknown” population. DNA was extracted from all samples and further processed for DArT sequencing to generate genome‐wide SNPs. Genetic clustering analyses such as DAPC, PCoA, and ADMIXTURE were used to determine the genetic proximity of the unknown population to the six provenances from the common garden. Clustering analyses of all individuals revealed that the unknown population is genetically related to southern populations. A focused analysis of only the southern and unknown populations showed that the unknown individuals formed a distinct cluster, suggesting they are not escapees but represent a natural population in the Southern California region. Although a spillage was not detected, these findings serve as a reminder for researchers to monitor their garden experiments to control escapees and reduce the possibility of a spillage occurring.

## Graphical abstract

In this study, we investigated a possible spillage of genetic material from an existing common garden experiment of monkeyflower and found no evidence of the unknown population of monkeyflower to be escapees but represent a natural population in that region.

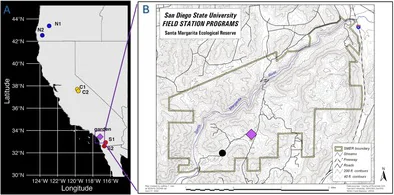

## 1 Introduction

Common garden experiments are a scientific approach for understanding the phenotypic traits of plant populations and how they are influenced by genetics and environmental conditions. Plants are taken from their natural habitats and are grown together under similar conditions, which can provide insights into research questions regarding climate change and environmental stressors (Berend et al. 2019; Schwinning et al. 2022). Some gardens are conducted indoors in greenhouses or growth chambers in controlled settings, making it easier to replicate and have precise control over various conditions found in natural environments. Common gardens can also be conducted outside, leaving them exposed to the elements under natural weather conditions (Jebali et al. 2025). They allow researchers to see how the plants grow without the use of artificial factors such as lighting and humidity. Moreover, several common gardens in different areas of species range (reciprocal transplant experiment) help determine local adaptation between plant populations by showing how they respond to environmental factors by growing them across latitudinal and elevational gradients (Rokaya et al. 2016). Further, for outcrossing species, common gardens can facilitate pollinators' access to the plants, enabling researchers to estimate individual plant fitness based on successful seed production and/or pollen dispersal (Souther et al. 2026).

While not entirely escape‐proof, indoor common gardens used for plant experiments provide substantially stronger containment compared to outdoor settings (Van Hengstum et al. 2012; Kim et al. 2018). One potential impact of outdoor common gardens is the plants escaping the garden and establishing themselves in the surrounding environment at the study site through means of seed and pollen dispersal. If not monitored, the plants may disrupt and even displace native species, thereby altering and complicating the restoration of native plant communities. This could lead to them taking up resources and modifying soil composition (Huxel 1999; Jordan et al. 2008). One other possibility that can occur as a result of escape is genetic spillage. This phenomenon occurs when external or foreign genotypes are introduced to the gene pool of the natural environment, altering the genetic patterns that have been established over time (Saba and Balwan 2023). Here, we define ‘genetic spillage’ as the unintended transfer of cultivated populations' alleles into the wild through both physical escape and establishment (through seed or vegetative dispersal), and pollen‐mediated gene flow. The physical escape and establishment refers to the dispersal of plant materials such as seeds, sections of plants, or the whole plant, beyond cultivated boundaries, which may result in self‐sustaining wild populations (Schafer et al. 2011). On the other hand, pollen‐mediated gene flow refers to the transfer of genes via pollen to reproductively compatible wild relatives, resulting in the stable incorporation of novel genes into wild genomes (e.g., Kang et al. 2021). While the former requires the survival and reproduction of escaped individuals, the latter can occur even when escaped plants cannot persist in the wild, through crossing with native plants.

Plant genetic spillage, particularly involving genetically modified (GM) crops, has been widely documented through both pollen and seed dispersal, resulting in gene flow into non‐GM and wild populations (Wang et al. 2016; Von Der Lippe and Kowarik 2007). Pollen‐mediated gene flow has been observed in several major crops, including oilseed rape, maize, potato, cotton, rice, and soybean, where transgenes have been shown to travel substantial distances and hybridize with wild relatives or conventional varieties (Liu et al. 2013; Saeglitz et al. 2000; Rong et al. 2010; Kang et al. 2021; Zhang et al. 2023; Wang et al. 2016). In seed‐mediated gene flow, seeds from GM crops can be unintentionally transported beyond cultivation sites, particularly along transportation routes, leading to the establishment of feral populations (Von Der Lippe and Kowarik 2007; Pascher et al. 2017). In contrast, studies documenting genetic spillage beyond GMOs are still limited, and the ones that exist have raised concerns. For instance, Whelan et al. (2006) examined morphological and genetic diversity in an urban population of *Grevillea macleayana* in southeastern Australia and noted that while native plants cultivated in gardens can enhance conservation, such plantings may also introduce gene flow that threatens the genetic integrity of nearby natural populations. Moreover, as large‐scale repositories of diverse plant material, botanical gardens and arboreta also function as global common gardens and may inadvertently serve as sources of plant escape. Recognizing this risk, the Public Gardens as Sentinels against Invasive Plants (PGSIP) initiative engages North American institutions in monitoring and genotyping plants that establish beyond cultivated collections (APGA 2026). Data from these observations are maintained in a public dashboard (PGSIP DATA DASHBOARD) to provide an early‐warning framework for identifying potentially invasive taxa before they spread. Despite growing awareness, plant escapees from common garden experiments remain poorly studied. Understanding these processes is essential, as experimental plantings can serve as unrecognized sources of genetic spillage, potentially influencing the genetic structure of surrounding natural populations.

According to Berend et al. (2019), outdoor common gardens require special care and monitoring to minimize negative impacts on the surrounding environment, especially if the site is protected. Despite extensive literature on how to conduct common garden experiments responsibly (Moloney et al. 2009; Berend et al. 2019), there is a lack of information available that assesses the impact of these experiments on the natural environment. When plants, or rather, their genotypes, escape from these gardens and are introduced into native or local populations, the long‐term effects are unknown and therefore must be monitored and assessed to mitigate any negative impacts they may have on native communities (Crozier and Schulte‐Hostedde 2015).

The scarlet monkeyflower, or *Erythranthe cardinalis* (syn. *Mimulus cardinalis* ) is a model system in evolutionary biology (Sheth et al. 2026). It maintains a mixed‐mating system characterized by both selfing and outcrossing. Recent studies indicate that even under environmental stressors such as extreme drought, populations often promote outcrossing by increasing nectar sugar content, rather than shifting toward a selfing syndrome (Wilborn‐Pilotte et al. 2025). This tendency toward outcrossing can be linked to its specialized pollination biology, as *E. cardinalis* is widely studied as a model for hummingbird pollination syndrome (Nelson et al. 2021). Its floral morphology is highly specialized for hummingbird attraction, with bright red petals that are attractive to avian pollinators but are less conspicuous to bees, which have limited sensitivity to red wavelengths (Wilbert et al. 1997). Although hummingbirds serve as its primary pollinators, rare yellow‐flowered morphs can arise through mutations in the *PELAN* gene that reduce anthocyanin production, potentially increasing visitation by bumblebees (Wenzell et al. 2023). In addition to pollinator‐mediated gene flow, seed dispersal may further influence population connectivity. Although direct studies of seed dispersal in *E. cardinalis* remain limited, evidence from its relative species, *E. guttatus* , suggests dispersal occurs primarily through water and wind as they have dust‐like seeds (Vickery 1968). Given that *E. cardinalis* typically occurs along stream margins (Jepson Flora Project 2023; Angert et al. 2011), hydrochory is likely its long‐distance dispersal mechanism.

**FIGURE 1:**
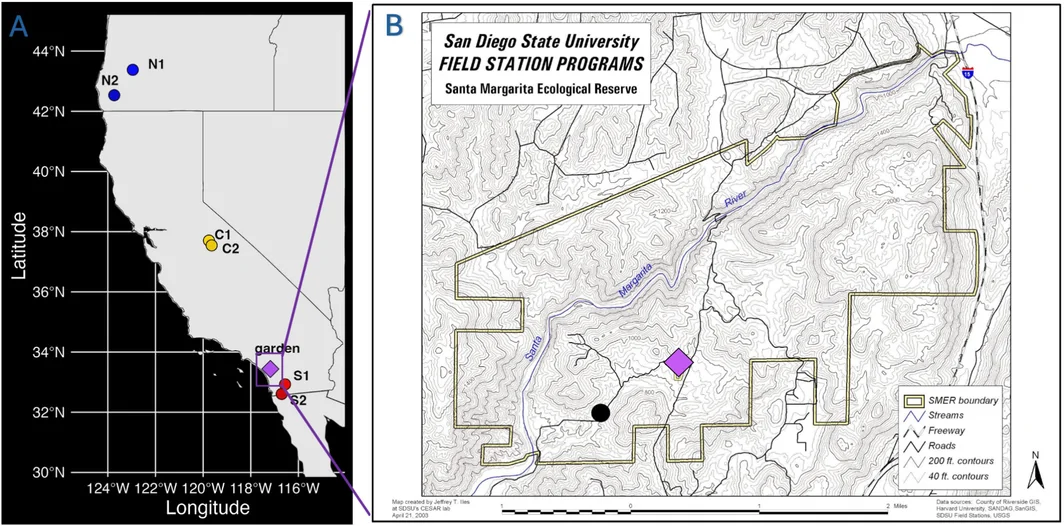
(A) Distribution map of *Erythranthe cardinalis* with population collection sites from previous research. The points correspond to six populations, two northern (blue), two central (yellow), and two southern (red). (B) Map of Santa Margarita Ecological Reserve. Colored dots correspond to the location of the southern garden (purple box) and the unknown population (black circle).

By using experimental common gardens and resurrection experiments, researchers investigate how *E. cardinalis* populations are adapting to environmental stressors such as climate change and how evolution can contribute to their persistence (Sheth et al. 2026). Here, we examine a common garden experiment that was established in 2023 near the southern range edge of its distribution to evaluate potential genetic spillage into the surrounding environment. This garden consisted of six populations of *E. cardinalis* from three regions across its geographic range: north (N1 and N2), central (C1 and C2), and south (S1 and S2; Figure 1), as described in Sheth et al. (2026). The main objective for establishing this common garden was to understand how wild populations of this species have responded to a multi‐year drought from 2010 to 2017 by comparing traits such as flowering time, plant size, and stomatal conductance between pre‐drought ancestors and post‐drought descendants (Sheth et al. 2026).

The garden under consideration is located in Temecula, California, at the Santa Margarita Ecological Reserve (SMER), managed by San Diego State University. This outdoor garden at SMER exposes the plants to drier environmental conditions near the southern range edge, making it an ideal and realistic environment to observe trait evolution in *E. cardinalis* . However, in the second year of the experiment (2024), a population of *E. cardinalis* that had not been detected by our team in previous years was spotted in proximity to the common garden, hereby referred to as the “unknown” population. Because common gardens are often established within the natural distribution of a species, nearby wild populations may be present. Consequently, monitoring surrounding populations for evidence of genetic spillage mediated by seed or pollen movement is essential to detect potential gene escape and to evaluate any unintended impacts on local populations (Crozier and Schulte‐Hostedde 2015).

This study seeks to determine if the unknown population is an escapee by conducting genetic analyses that evaluate the similarities and differences between the six experimental populations in the garden and the unknown population found nearby, allowing us to subsequently assess a potential spillage event. We expect that two different genetic signatures will be detected depending on whether a gene spillage has occurred from seed (similar genotypes to any of the six populations established in the common garden) or pollen movement (introgression between any of our six populations at the garden and the native population). Additionally, if no genetic spillage has occurred, we would expect the “unknown” population to have a distinct genetic profile from the six common garden populations, given documented genetic differentiation in the system (Paul et al. 2011).

## 2 Materials and Methods

### 2.1 Study System

Scarlet monkeyflower ( *Erythranthe cardinalis* syn. *Mimulus cardinalis* ) is a perennial herb native to the Western United States and Baja California (Fraga 2018). Its distribution runs from southern Oregon, throughout California, and into northern Baja California, where it grows in riparian habitats along seeps and stream banks (Angert and Schemske 2005). This species has been a subject of many ecological studies investigating its geographical range limits, local adaptation, and response to climate change (i.e., Angert and Schemske 2005; Angert et al. 2011; Sheth et al. 2026; Waits et al. 2025).

### 2.2 Study Site

This study focuses on a garden established in SMER, Temecula, CA, near the southern range edge of *E. cardinalis*. This garden is part of a larger experiment testing drought response in this species (see Sheth et al. 2026). The garden consists of six populations of *E. cardinalis* that have been used in previous studies investigating the response to climate change of this species (Sheth and Angert 2016; Vtipil and Sheth 2020; Wooliver et al. 2020; Albano et al. 2025; Sheth et al. 2026; Waits et al. 2025). The seeds of these populations have been collected from three regions across the species' distribution in the California Floristic Province: north (N1, N2), central (C1, C2), and south (S1, S2) (Table 1, Figure 1). This experiment was carried out from 2023 to 2025 and only focal plants carefully crossed in the greenhouse were transplanted into the common garden and kept alive during the length of the experiment, that is, no new seedlings were allowed to germinate within the garden (see more details in Sheth et al. 2026; Waits et al. 2025).

**TABLE 1:** Information for sampled populations used in this study, including population ID, locality, and geographic coordinates of the source populations, such as latitude, longitude, and elevation. Populations N1, N2, and S2 were collected from sites managed by the Bureau of Land Management, Idleyld Park, Oregon, Wilderville, Oregon, and Dulzura, California, respectively. C1 and C2 were collected from Yosemite National Park, and S1 was collected from Cuyamaca Rancho State Park. The unknown population was sampled from Santa Margarita Ecological Reserve.

Population ID	Locality	Latitude (°N)	Longitude (°W)	Elevation (m)
N1	Idleyld Park	43.37876	−122.95207	295
N2	Wilderville	42.53529	−123.73016	914
C1	Yosemite National Park	37.70377	−119.75363	1316
C2	Yosemite National Park	37.54576	−119.64152	1228
S1	Cuyamaca Rancho State Park	32.92788	−116.56019	1252
S2	Dulzura	32.60831	−116.70098	252
Unknown	Santa Margarita Ecological Reserve	33.43369	−117.18702	183

### 2.3 Sample Collection

To determine relatedness among the garden populations and the unknown population of *E. cardinalis* , leaf tissue from 9 to 15 individuals per population was collected from the garden. Individuals were also collected from the unknown population, with a total of 84 samples (N1 = 9, N2 = 11, C1 = 13, C2 = 15, S1 = 11, S2 = 11, unknown = 14). In an attempt to avoid collecting ramets of the same genet from the unknown population, we sampled plants at least two meters apart, except for three plants. Samples were collected at the end of August in 2024, just before plants from the garden began to wither.

### 2.4 DNA Extraction and Sequencing

Genomic DNA was extracted from the plant tissues using the DNeasy Plant Mini Kit by Qiagen. After confirming high molecular weight DNA presence using gel electrophoresis, all 84 samples were submitted to Diversity Arrays Technology (DArT (Diversity Arrays Technology Pty Ltd., Canberra, Australia)) for sequencing. DArTseq is a technology that combines genome complexity reduction methods with next‐generation sequencing on Illumina platforms to genotype thousands of single nucleotide polymorphisms (SNPs) (Jaccoud 2001; Bocianowski et al. 2023). Genome complexity is reduced through the use of specific restriction endonucleases that preferentially target low‐copy DNA regions, enabling efficient and reproducible SNP discovery and genotyping (Jaccoud 2001). The DArTseq approach is initially de novo, enabling the identification of SNPs without needing a reference genome. However, the reference genome of *E. guttata* and *E. laciniata* species was subsequently used for physical mapping and determination of chromosomal positions of the SNPs.

Genomic DNA extraction and quality assessment were performed by the authors. Subsequent library preparation, genome complexity reduction, next‐generation sequencing, SNP calling, and initial marker filtering were conducted by DArT using the DArTsoft14 bioinformatics pipeline (Kilian et al. 2012; Chevrier et al. 2024).

The DArTseq data then underwent critical filtering to remove SNP loci that would not be included in the analysis, such as those with more than 10% missing data (90% call rate), < 100% reproducibility, departures from Hardy–Weinberg equilibrium, monomorphic loci, and secondaries (multiple SNP loci present within one sequenced fragment which could be linked). The data filtering was carried out using the “gl.filter” function for all the above mentioned criteria and was followed by conversion into file formats suitable for conducting a genetic analysis. Both were performed using the dartR package (Gruber et al. 2018) in RStudio (RStudio Team 2015). Following all filtering steps, a total of 6345 SNPs were retained for downstream analyses.

### 2.5 Genetic Analysis

To determine if the unknown population of *E. cardinalis* is an escapee, genetic analyses were performed using three distinct methods. A Principal Coordinates Analysis (PCoA) was performed using the gl.pcoa.plot function in the dartR package (Gruber et al. 2018). This approach helped visualize the differences in variation among the populations. To infer genetic clustering among the populations, Discriminant Analysis of Principal Components (DAPC) was performed using the adegenet package (Jombart 2008) in RStudio (RStudio Team 2015). The xvalDapc() function was used for a cross‐validation to identify the appropriate number of retained principal components. If populations clustered together, we performed a sub PcoA or sub DAPC analysis to increase the within‐cluster resolution. The third method, ADMIXTURE, is a model‐based maximum likelihood approach that was used to estimate the genetic structure and individual ancestral proportions of the populations (Alexander et al. 2009). Analyses were run from K = 1–10 with 3 replicates for each K value with different random seeds. The best K was chosen following the default cross‐validation (CV) method implemented in ADMIXTURE, where CV error (measures how well the inferred population structure model predicts training dataset) was compared across the runs, and the model with the lowest CV error was chosen as the best supported model. The ancestral proportions for each individual from a gene pool (Q estimates obtained from ADMIXTURE) were plotted and visualized using bar plots in MS Excel for clarity and ease of ordering.

Using the stamppFst command in the R package adegenet (Jombart 2008), *F*_ST_ values were calculated to determine differentiation among all populations of *E. cardinalis* to further quantify whether the unknown population is an escapee (genetic spillage) from our common garden experiment. A genetic diversity analysis was performed in RStudio (RStudio Team 2015) using the function gl.report.heterozygosity from the dartR package (Gruber et al. 2018) to assess expected (He), observed (Ho) heterozygosity, and inbreeding coefficient (FIS). Positive FIS values indicated inbreeding, and negative values indicated outbreeding. Although the genetic diversity and inbreeding metrics are not directly indicative of population origin, if the potential escapees represent a recently founded population established from a few seeds originating from the common garden, they would exhibit low genetic diversity and elevated inbreeding due to founder effects and limited opportunities for gene flow (Hartl and Clark 2007) in the surrounding area. Therefore, estimates of heterozygosity and inbreeding remain informative and were included in our analyses.

## 3 Results

**FIGURE 2:**
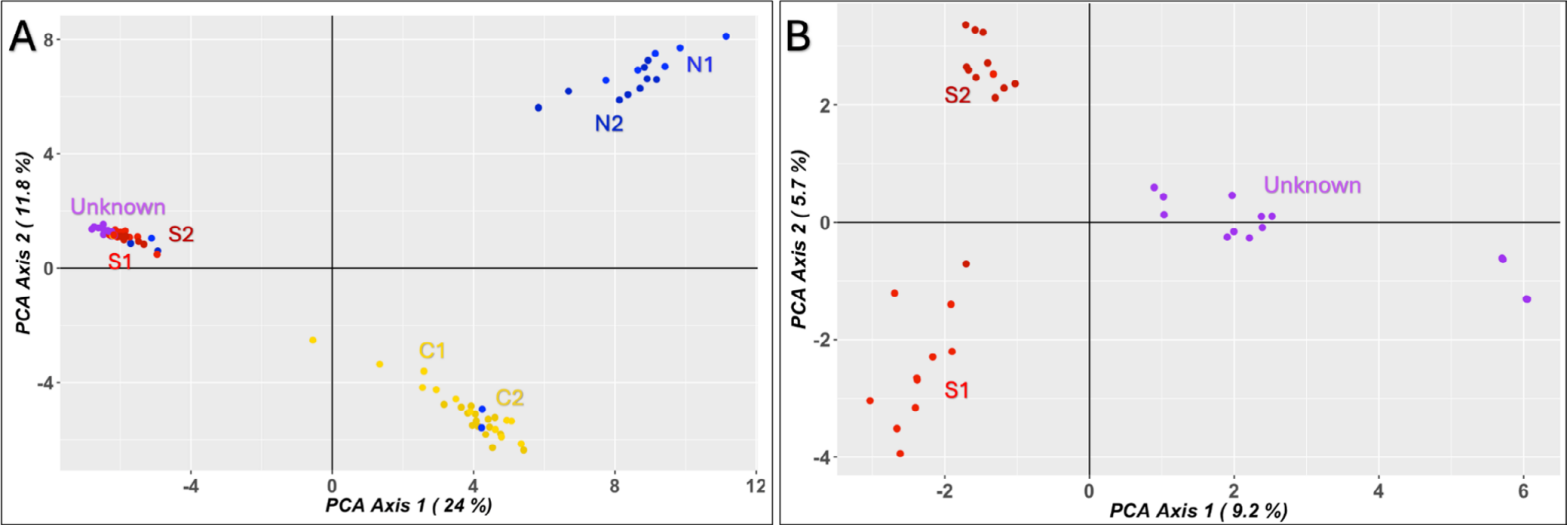
(A) PCoA scatterplot showing differentiation among the seven populations of *Erythranthe cardinalis* . Northern populations in blue, central populations in yellow, southern populations in red, and the unknown population in purple. (B) PCoA scatterplot showing differentiation between southern populations and the unknown population of *E. cardinalis* .

A total of 48,919 unfiltered SNPs were generated from DArTseq. After undergoing filtering to remove those with more than 10% missing data (90% call rate), < 100% reproducibility, departures from Hardy–Weinberg equilibrium, monomorphs and secondaries, 6345 binary SNPs were retained for the following genetic analyses. The PCoA, based on a 90% SNP call rate for all seven populations, revealed the pattern of genetic structure within the populations. The first two principal coordinates (PC1 and PC2) explained 23.3% and 12% of the total genetic variation, respectively (Figure 2A). The PCoA plot revealed distinct clustering among populations from the north, central, and south, while the unknown individuals clustered closely with the southern populations. The two populations from the north, central, and south each formed a tight cluster within their respective regions. The southern populations appeared more distantly related to the northern than to the central populations. The unknown population was more similar to the southern populations.

A sub PCoA based on a 90% SNP call rate for just the southern and unknown populations revealed genetic differentiation between these populations (Figure 2B). The first two principal coordinates (PC1 and PC2) explained 9.2% and 5.7% of the total genetic variation, respectively. The PCoA plot revealed that the southern populations were clearly separated from the unknown individuals.

**FIGURE 3:**
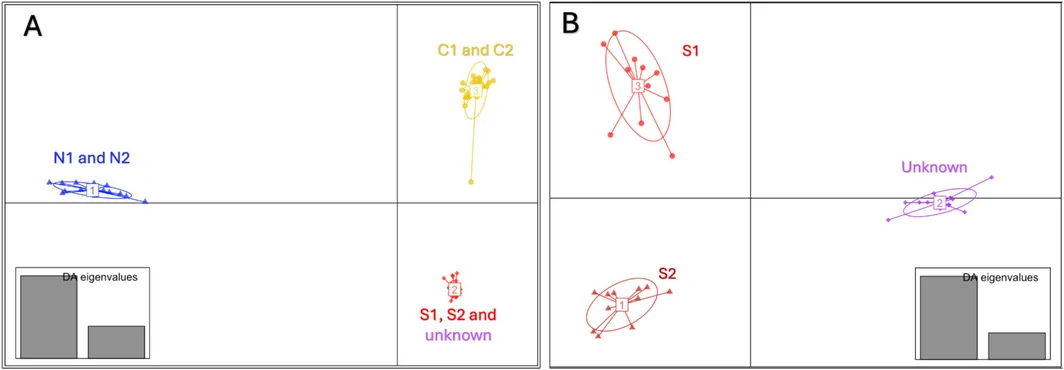
(A) DAPC scatterplot showing 3 genetic clusters among the seven populations of *Erythranthe cardinalis* . Northern clusters in blue, central clusters in yellow, and southern clusters with unknown clusters in red. (B) DAPC scatterplot showing 3 genetic clusters among southern populations and the unknown population of *E. cardinalis* .

The DAPC plot based on a 90% SNP call rate (Figure 3A) revealed clear clustering of individuals into three groups. Populations from the same geographic region clustered together, with N1 and N2 forming one group, and S1, S2, and C1, C2 forming the other clusters. The unknown population clustered along with the S1 and S2 groups. The northern populations appear more distantly related to the central and southern populations. The unknown population seems to be genetically similar to the southern populations.

A sub DAPC based on a 90% SNP call rate for just the south and unknown populations revealed clear genetic differentiation between southern populations (Figure 3B). The analysis revealed three clusters, where S1 and S2 formed two separate clusters, and the unknown population separated into its own cluster.

**FIGURE 4:**
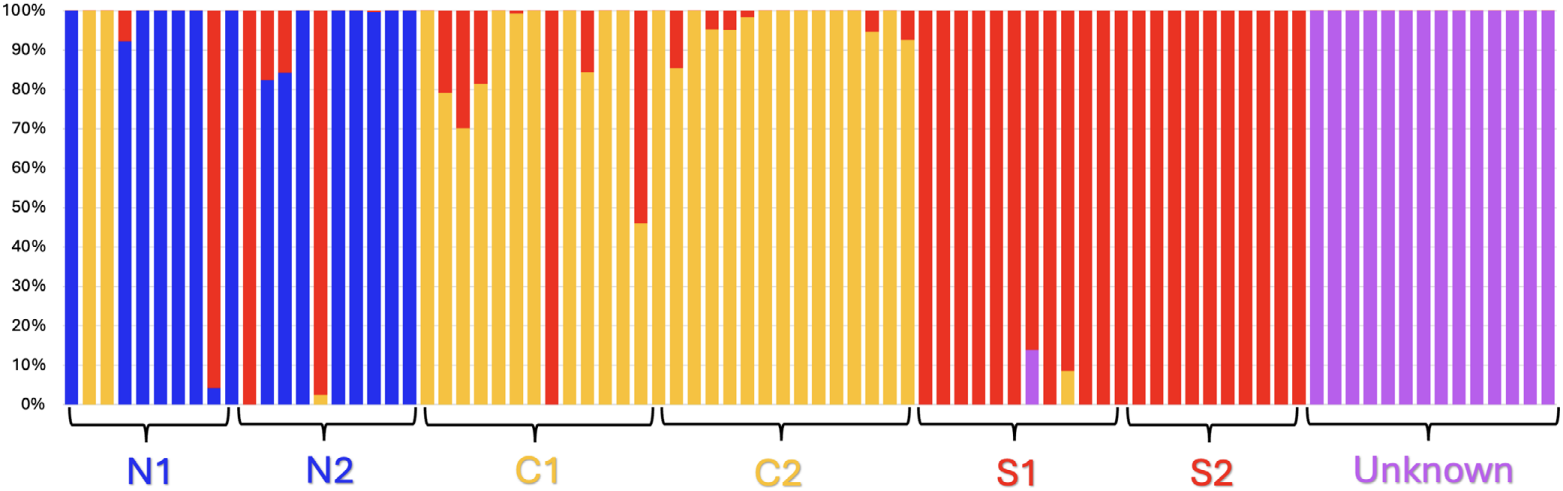
ADMIXTURE plot showing four distinct clusters among the seven populations of *Erythranthe cardinalis* .

ADMIXTURE analysis revealed four distinct genetic clusters among the seven populations of *E. cardinalis* (Figure 4). The clustering patterns largely corresponded to geographic regions, with individuals from northern populations (N1, N2) predominantly assigned to one cluster, central (C1, C2) populations forming one cluster, and southern populations (S1, S2) assigned to a separate cluster. The unknown population formed its own distinct cluster. All populations except the unknown population showed evidence of admixture, with individuals displaying partial ancestry from multiple clusters. One individual in S1 showed partial ancestry derived from the wild population gene pool.

Pairwise *F*_ST_ values among the seven populations ranged from 0.037 to 0.321 (Table 2). Populations C1 and C2 showed the lowest differentiation, while the unknown and N1 were the most distinct. The overall *F*_ST_indicates moderate genetic differentiation among populations, and all values were significantly different from zero.

**TABLE 2:** Pairwise *F*_ST_ showing differentiation for all seven populations of *Erythranthe cardinalis* .

	N1	N2	C1	C2	S1	S2	Unknown
N1	x						
N2	0.037	x					
C1	0.131	0.153	x				
C2	0.148	0.18	0.029	x			
S1	0.299	0.272	0.202	0.260	x		
S2	0.3	0.274	0.201	0.262	0.037	x	
Unknown	0.321	0.296	0.226	0.284	0.068	0.064	x

The observed heterozygosity (Ho) ranged from 0.043 to 0.104, while the expected heterozygosity (He) ranged from 0.041 to 0.096 across populations (Table 3). All south populations, including the unknown population, showed the lowest genetic diversity, with S2 being the lowest. N2 had the highest genetic diversity. In general, Ho > He, suggesting outcrossing among the populations.

**TABLE 3:** Expected heterozygosity (He), observed heterozygosity (Ho), and inbreeding values for all seven populations of *Erythranthe cardinalis* .

Population	Expected heterozygosity (He)	Observed heterozygosity (Ho)	Inbreeding (FIS)
N1	0.093	0.085	0.133
N2	0.096	0.104	−0.026
C1	0.088	0.095	−0.040
C2	0.086	0.093	−0.049
S1	0.044	0.046	0.003
S2	0.041	0.043	−0.014
Unknown	0.048	0.044	0.110

## 4 Discussion

Assessing the genetic structure of the unknown population located near the common garden experiment is crucial for determining whether genetic spillage has occurred and if the local populations are under threat. The DAPC and PCoA analyses showed similar genetic patterns, revealing genetic similarity between the southern and unknown populations, indicating they are local to the southern region. Furthermore, a sub‐analysis revealed a genetic distinction between the unknown and southern populations, suggesting the unknown population is natural and not an escapee. We found no evidence for genetic spillage from the common garden either through seed movement or pollen‐mediated introgression.

Both DAPC and PCoA analyses displayed the six populations, two north, two central, and two south, as distinct clusters separated by their area of origin. The northern and southern parts of California are known to differ in climatic conditions, with the north experiencing more precipitation and cooler temperatures, and the south experiencing hotter and drier environments (He and Gautam 2016). Given the difference in climatic conditions within those regions, these populations may experience different selective pressures, which could have contributed to their genetic differentiation. A recent study by Albano et al. (2025) also reported differences in traits such as specific leaf area (SLA) and leaf dry matter content (LDMC), which are indicators of adaptive plant functional responses to environmental variation, among samples from different parts of the *E. cardinalis* range. Additionally, genetic differentiation among these populations may also result from neutral processes such as genetic drift and demographic divergence arising from long‐term geographic separation (Holsinger and Weir 2009). The DAPC and PCoA analyses also revealed the connection between the south and unknown populations, suggesting the latter to have originated from the southern region rather than the northern or central regions. Pairwise *F*_ST_values shown in Table 2 displayed low genetic differentiation between the unknown population and the two southern populations (S1, S2), further confirming genetic similarity. However, sub DAPC and PCoA analyses showed genetic differentiation between the unknown and southern populations, suggesting an escape from the garden to be unlikely.

The unknown population exhibited no evidence of introgression from the garden. If genetic spillage had occurred via seed movement, we would expect individuals in the unknown population to show complete ancestry assignment to one of the six common garden population pools. Similarly, pollen‐mediated gene flow would be expected to produce individuals with partial ancestry derived from one or more populations' gene pools. Together, the ADMIXTURE results provide no support for genetic spillage via either seed dispersal or pollen‐mediated gene flow. The absence of admixture within the unknown population may reflect restricted gene flow resulting from geographic, reproductive, or ecological barriers that limit connectivity with other populations as shown in other groups (Flores‐Renteria et al. 2025). Given that the unknown population appeared small in size in the wild, it is also possible that it recently underwent a genetic bottleneck, leading to reduced genetic diversity that could obscure signals of historical admixture (Ong et al. 2024). Alternatively, there is a possibility that the admixture signals are weak due to limited sampling or low genetic marker resolution.

In contrast, the remaining six populations displayed clear evidence of admixture among some individuals. This pattern might be indicative of gene flow among these populations, potentially facilitated by overlapping flowering periods, shared pollinators, or anthropogenic factors (Paul et al. 2011; Oliveira et al. 2021). Hummingbirds, including Anna's hummingbird, *Calypte anna* , are *E. cardinalis'* primary pollinators and are common all year round but are most abundant during late fall to winter in the chaparral, around the same time as the plant's bloom time, which is from May to September (Nesom 2014; Battey 2019). When the birds visit the flowers, the pollen they acquire while feeding may remain on the bird's beak and head, and potentially be transferred to another flower from a different population the bird visits (Castellanos et al. 2003). Alternatively, this admixture pattern could indicate incomplete lineage sorting, convergence to similar genetic variations, or just statistical artifacts due to the limited number of samples (Ramadoss et al. 2025). Interestingly, a single individual from the S1 population was observed to share ancestral proportions (~13.8%) with the wild population, indicating possible shared ancestry between the two populations or limited gene flow from the wild population. This is possible given that they are less than 80 km apart. Further coalescent‐based analyses using additional samples and higher‐density genomic data would help clarify the underlying processes.

We found that there is higher genetic diversity in the northern populations than in the southern populations of *E. cardinalis* . This is contradictory to the hypothesis of a recent northward expansion for this species, expecting higher genetic diversity in the south (Sheth and Angert 2018). A recent analysis of monkeyflowers by Nelson et al. (2021) revealed that northern and southern populations of *E. lewisii* (formerly known as *Mimulus lewisii* ) form genetically distinct clades, and northern populations have higher genetic diversity than the southern populations. Therefore, different evolutionary histories might be at play for these species.

Pairwise *F*_ST_ values among the seven populations ranged from 0.037 to 0.321, indicating low to moderate genetic differentiation. This range is comparable to findings from a previous study on the native subalpine plant *E. lewisii* , which reported weak genetic differentiation between foreland populations (*F*_ST_ = 0.035) based on ddRADseq variable sites (Urquhart‐Cronish et al. 2025).

Previous research using these same populations has described important aspects regarding survival and genetic variation in this species, revealing strong fitness differences between populations, especially during times of drought. A study by Sheth et al. (2026) found that southern populations produced more reproductive structures and had the tallest maximum stem height compared to the northern and central populations, with northern populations being the shortest in the common garden at SMER. Additionally, when comparing performance among three gardens located in the northern, central, and southern distribution of *E. cardinalis*, the study found that across all populations and gardens, plants that flowered earliest had the highest fitness, especially in the southern garden, which is consistent with escape strategies to overcome intense temperatures in that region (Anstett et al. 2021). Other studies have shown that southern populations exhibit greater genetic variation and even higher photosynthetic growth rates, enabling them to adapt to climate change, while northern and central populations have lower fitness in the southern region and may struggle to survive (Sheth and Angert 2016; Muir and Angert 2017; Muir et al. 2021). This suggests that there is a low likelihood of northern and central plants establishing in the southern region if they were to escape. However, even under climate change, northern and central populations may still have the ability to adapt, which underscores the need for long‐term monitoring in the field to evaluate genetic spillage in this and similar studies.

## Author Contributions

**Lydia Duran:** formal analysis (equal), investigation (equal), writing – original draft (equal). **Niveditha Ramadoss:** formal analysis (equal), writing – original draft (equal). **Seema Nayan Sheth:** conceptualization (equal), funding acquisition (equal), investigation (equal), supervision (equal), writing – review and editing (equal). **Lluvia Flores‐Rentería:** conceptualization (equal), formal analysis (equal), funding acquisition (equal), investigation (equal), supervision (lead), writing – original draft (equal).

## Funding

This work was supported by the NSF DEB grants: 2131815, 2131819, and the USDA's National Institute of Food and Agriculture's From Learning to Leading: Cultivating the Next Generation of Diverse Food and Agriculture Professionals Program (NEXTGEN) grant no. 2023‐70440‐40156/project accession no. 1030734, and a Research Capacity Fund (HATCH) project award (7002993) from the US Department of Agriculture's National Institute of Food and Agriculture.

## Conflicts of Interest

The authors declare no conflicts of interest.

## Data Availability

The genomic SNP data is available in Dryad at https://doi.org/10.5061/dryad.gxd25481v.

## References

[ece374226-bib-0001] Albano, L. J. , R. A. Bingham , S. Correa , et al. 2025. “Range‐Wide Responses to an Extreme Heat Event in *Mimulus cardinalis*.” bioRxiv. 10.1101/2025.05.01.651703. PMC12918904 41536030

[ece374226-bib-0002] Alexander, D. H. , J. Novembre , and K. Lange . 2009. “Fast Model‐Based Estimation of Ancestry in Unrelated Individuals.” Genome Research 19: 1655–1664. 10.1101/gr.094052.109. 19648217 PMC2752134

[ece374226-bib-0003] Angert, A. L. , and D. W. Schemske . 2005. “The Evolution of Species' Distributions: Reciprocal Transplants Across the Elevation Ranges of *Mimulus cardinalis* and *M. lewisii* .” Evolution 59: 1671–1684. 10.1111/j.0014-3820.2005.tb01817.x. 16329239

[ece374226-bib-0004] Angert, A. L. , S. N. Sheth , and J. R. Paul . 2011. “Incorporating Population‐Level Variation in Thermal Performance Into Predictions of Geographic Range Shifts.” Integrative and Comparative Biology 51: 733–750. 10.1093/icb/icr048. 21705795

[ece374226-bib-0005] Anstett, D. N. , H. A. Branch , and A. L. Angert . 2021. “Regional Differences in Rapid Evolution During Severe Drought.” Evolution Letters 5: 130–142. 10.1002/evl3.218. 33868709 PMC8045920

[ece374226-bib-0006] APGA . 2026. “Public Gardens as Sentinels Against Invasive Plants Initiative.” American Public Gardens Association Dashboard. https://members.publicgardens.org/grow‐your‐garden/protect/pgsaip/.

[ece374226-bib-0007] Battey, C. J. 2019. “Ecological Release of the Anna's Hummingbird During a Northern Range Expansion.” American Naturalist 194: 306–315. 10.1086/704249. 31553208

[ece374226-bib-0008] Berend, K. , K. Haynes , and C. M. MacKenzie . 2019. “Common Garden Experiments as Dynamic Tool for Ecological Studies of Alpine Plants and Communities in Northeastern North America.” Rhodora 121: 174. 10.3119/18-16.

[ece374226-bib-0009] Bocianowski, J. , A. Tomkowiak , M. Bocianowska , and A. Sobiech . 2023. “The Use of DArTseq Technology to Identify Markers Related to the Heterosis Effects in Selected Traits in Maize.” Current Issues in Molecular Biology 45, no. 4: 2644–2660. 10.3390/cimb45040173. 37185697 PMC10136425

[ece374226-bib-0010] Castellanos, M. C. , P. Wilson , and J. D. Thomson . 2003. “Pollen Transfer by Hummingbirds and Bumblebees, and the Divergence of Pollination Modes in Penstemon.” Evolution 57: 2742–2752. 10.1111/j.0014-3820.2003.tb01516.x. 14761053

[ece374226-bib-0011] Chevrier, T. , D. A. Cowart , A. E. Nieblas , et al. 2024. “Population Structure of the Swordfish, *Xiphias gladius* , Across the Indian Ocean Using Next‐Generation Sequencing.” ICES Journal of Marine Science 82: fsae179. 10.1093/icesjms/fsae179.

[ece374226-bib-0012] Crozier, G. K. D. , and A. I. Schulte‐Hostedde . 2015. “Towards Improving the Ethics of Ecological Research.” Science and Engineering Ethics 21: 577–594. 10.1007/s11948-014-9558-4. 24903671 PMC4430594

[ece374226-bib-0013] Flores‐Renteria, L. , A. McElwee‐Adame , N. Ramadoss , M. Gonzalez‐Elizondo , R. Sniezko , and M. S. Gonzalez‐Elizondo . 2025. “Multidirectional Hybridization Challenges the Species Barriers in North American Arbutus (Ericaceae).” Global Ecology and Conservation 59: e03572. 10.1016/j.gecco.2025.e03572.

[ece374226-bib-0014] Fraga, N. S. 2018. “ *Erythranthe cardinalis* , in Jepson Flora Project (eds.) Jepson eFlora, Revision 6.”

[ece374226-bib-0015] Gruber, B. , P. J. Unmack , O. F. Berry , and A. Georges . 2018. “Dartr: An r Package to Facilitate Analysis of SNP Data Generated From Reduced Representation Genome Sequencing.” Molecular Ecology Resources 18: 691–699. 10.1111/1755-0998.12745. 29266847

[ece374226-bib-0016] Hartl, D. L. , and A. G. Clark . 2007. Principles of Population Genetics. 4th ed. Sinauer Associates.

[ece374226-bib-0017] He, M. , and M. Gautam . 2016. “Variability and Trends in Precipitation, Temperature and Drought Indices in the State of California.” Hydrology 3, no. 2: 14. 10.3390/hydrology3020014.

[ece374226-bib-0018] Holsinger, K. , and B. Weir . 2009. “Genetics in Geographically Structured Populations: Defining, Estimating and Interpreting *F* _ST_ .” Nature Reviews. Genetics 10: 639–650. 10.1038/nrg2611. PMC4687486 19687804

[ece374226-bib-0019] Huxel, G. R. 1999. “Rapid Displacement of Native Species by Invasive Species: Effects of Hybridization.” Biological Conservation 89: 143–152. 10.1016/S0006-3207(98)00153-0.

[ece374226-bib-0020] Jaccoud, D. 2001. “Diversity Arrays: A Solid State Technology for Sequence Information Independent Genotyping.” Nucleic Acids Research 29: e25. 10.1093/nar/29.4.e25. 11160945 PMC29632

[ece374226-bib-0021] Jebali, A. , H. Kaur , H. Martinez , et al. 2025. “Two Years of Outdoor Cultivation in Alternate Climates Produces Little Divergence in the Productivity of *Nannochloropsis* .” Science of the Total Environment 981: 179587. 10.1016/j.scitotenv.2025.179587. 40328070

[ece374226-bib-0022] Jepson Flora Project . 2023. “ *Erythranthe cardinalis* .” In *Jepson eFlora* (Revision 12). The Jepson Herbarium, University of California, Berkeley. https://ucjeps.berkeley.edu/eflora/eflora_display.php?tid=25147.

[ece374226-bib-0023] Jombart, T. 2008. “Adegenet: A R Package for the Multivariate Analysis of Genetic Markers.” Bioinformatics 24: 1403–1405. 10.1093/bioinformatics/btn129. 18397895

[ece374226-bib-0024] Jordan, N. R. , D. L. Larson , and S. C. Huerd . 2008. “Soil Modification by Invasive Plants: Effects on Native and Invasive Species of Mixed‐Grass Prairies.” Biological Invasions 10: 177–190. 10.1007/s10530-007-9121-1.

[ece374226-bib-0025] Kang, H.‐G. , O.‐C. Chung , T.‐W. Bae , et al. 2021. “Pollen‐Mediated Flow of Bar Gene in Transgenic Herbicide‐Resistant Turf Grass *Zoysia japonica* .” Plant Biotechnology Reports 15: 241–250. 10.1007/s11816-021-00667-4.

[ece374226-bib-0026] Kilian, A. , P. Wenzl , E. Huttner , et al. 2012. “Diversity Arrays Technology: A Generic Genome Profiling Technology on Open Platforms.” In Data Production and Analysis in Population Genomics: Methods and Protocols, 67–89. Humana Press. 10.1007/978-1-61779-870-2_5. 22665276

[ece374226-bib-0027] Kim, D.‐S. , I. Song , and K. Ko . 2018. “Low Risk of Pollen‐Mediated Gene Flow in Transgenic Plants Under Greenhouse Conditions.” Horticulture, Environment, and Biotechnology 59: 723–728. 10.1007/s13580-018-0074-3.

[ece374226-bib-0028] Liu, Y. , W. Wei , K. Ma , J. Li , Y. Liang , and H. Darmency . 2013. “Consequences of Gene Flow Between Oilseed Rape ( *Brassica napus* ) and Its Relatives.” Plant Science 211: 42–51. 10.1016/j.plantsci.2013.07.002. 23987810

[ece374226-bib-0029] Moloney, K. A. , C. Holzapfel , K. Tielbörger , F. Jeltsch , and F. M. Schurr . 2009. “Rethinking the Common Garden in Invasion Research.” Perspectives in Plant Ecology, Evolution and Systematics 11: 311–320. 10.1016/j.ppees.2009.05.002.

[ece374226-bib-0030] Muir, C. D. , and A. L. Angert . 2017. “Grow With the Flow: A Latitudinal Cline in Physiology Is Associated With More Variable Precipitation in *Erythranthe cardinalis* .” Journal of Evolutionary Biology 30: 2189–2203. 10.1111/jeb.13184. 28977720

[ece374226-bib-0031] Muir, C. D. , C. L. Van Den Elzen , and A. L. Angert . 2021. “Selection on Early Survival Does Not Explain Germination Rate Clines in *Mimulus cardinalis* (Phrymaceae).” *bioRxiv*, pp.2021‐12. 10.1101/2021.12.14.472651. 36317645

[ece374226-bib-0032] Nelson, T. C. , A. M. Stathos , D. D. Vanderpool , F. R. Finseth , Y. Yuan , and L. Fishman . 2021. “Ancient and Recent Introgression Shape the Evolutionary History of Pollinator Adaptation and Speciation in a Model Monkeyflower Radiation (*Mimulus Section Erythranthe*) (A Buerkle, ed.).” PLoS Genetics 17: e1009095. 10.1371/journal.pgen.1009095. 33617525 PMC7951852

[ece374226-bib-0033] Nesom, G. L. 2014. “Taxonomy of *Erythranthe Sect. Erythranthe* (Phrymaceae).” Phyton 31: 1–41.

[ece374226-bib-0034] Oliveira, G. C. X. , I. C. G. Vieira , and R. L. Tremblay . 2021. “Gene Flow Among Wild and Domesticated Plant Species in the Neotropical Region.” Frontiers in Ecology and Evolution 9: 799071. 10.3389/fevo.2021.799071.

[ece374226-bib-0035] Ong, H. G. , E. Jung , Y.‐I. Kim , et al. 2024. “Population Connectivity and Size Reductions in the Anthropocene: The Consequence of Landscapes and Historical Bottlenecks in White Forsythia Fragmented Habitats.” BMC Ecology and Evolution 24: 123. 10.1186/s12862-024-02308-0. 39390358 PMC11465745

[ece374226-bib-0036] Pascher, K. , C. Hainz‐Renetzeder , G. Gollmann , and G. M. Schneeweiss . 2017. “Spillage of Viable Seeds of Oilseed Rape Along Transportation Routes: Ecological Risk Assessment and Perspectives on Management Efforts.” Frontiers in Ecology and Evolution 5: 104. 10.3389/fevo.2017.00104.

[ece374226-bib-0037] Paul, J. R. , S. N. Sheth , and A. L. Angert . 2011. “Quantifying the Impact of Gene Flow on Phenotype‐Environment Mismatch: A Demonstration With the Scarlet Monkeyflower *Mimulus cardinalis* .” American Naturalist 178: S62–S79. 10.1086/661781. 21956093

[ece374226-bib-0038] Ramadoss, N. , S. Steele , and L. Flores‐Renteria . 2025. “Prickly Problems: Cylindropuntia's Low Genetic Diversity Despite Inbreeding Avoidance.” Ecology and Evolution 15: e71213. 10.1002/ece3.71213. 40235723 PMC11997463

[ece374226-bib-0039] Rokaya, M. B. , T. Dostálek , and Z. Münzbergová . 2016. “Plant‐Herbivore Interactions Along Elevational Gradient: Comparison of Field and Common Garden Data.” Acta Oecologica 77: 168–175. 10.1016/j.actao.2016.10.011.

[ece374226-bib-0040] Rong, J. , Z. Song , T. J. De Jong , et al. 2010. “Modelling Pollen‐Mediated Gene Flow in Rice: Risk Assessment and Management of Transgene Escape.” Plant Biotechnology Journal 8: 452–464. 10.1111/j.1467-7652.2009.00488.x. 20132516

[ece374226-bib-0041] RStudio Team . 2015. RStudio: Integrated Development for R. RStudio Inc. http://www.rstudio.com/.

[ece374226-bib-0042] Saba, N. , and W. K. Balwan . 2023. “Genetic Pollution: A Safe or Risky Bet?” Scholars Academic Journal of Biosciences 11: 159–162. 10.36347/sajb.2023.v11i04.004.

[ece374226-bib-0043] Saeglitz, C. , M. Pohl , and D. Bartsch . 2000. “Monitoring Gene Flow From Transgenic Sugar Beet Using Cytoplasmic Male‐Sterile Bait Plants.” Molecular Ecology 9: 2035–2040. 10.1046/j.1365-294X.2000.01109.x. 11123616

[ece374226-bib-0044] Schafer, M. G. , A. A. Ross , J. P. Londo , et al. 2011. “The Establishment of Genetically Engineered Canola Populations in the U.S.” PLoS One 6, no. 10: e25736. 10.1371/journal.pone.0025736. 21998689 PMC3187797

[ece374226-bib-0045] Schwinning, S. , C. J. Lortie , T. C. Esque , and L. A. DeFalco . 2022. “What Common‐Garden Experiments Tell Us About Climate Responses in Plants.” Journal of Ecology 110: 986–996. 10.1111/1365-2745.13887.

[ece374226-bib-0046] Sheth, S. N. , L. J. Albano , C. Blanchard , et al. 2026. “Evolutionary Responses to Historic Drought Across the Range of Scarlet Monkeyflower.” American Naturalist 207: 135–155. 10.1086/738434. 41418304

[ece374226-bib-0047] Sheth, S. N. , and A. L. Angert . 2016. “Artificial Selection Reveals High Genetic Variation in Phenology at the Trailing Edge of a Species Range.” American Naturalist 187: 182–193. 10.1086/684440. 26807746

[ece374226-bib-0048] Sheth, S. N. , and A. L. Angert . 2018. “Demographic Compensation Does Not Rescue Populations at a Trailing Range Edge.” Proceedings of the National Academy of Sciences of the United States of America 115, no. 10: 2413–2418. 10.1073/1715899115. 29463711 PMC5878003

[ece374226-bib-0049] Souther, S. , C. E. Aslan , K. C. Grady , and K. A. Haubensak . 2026. “Evaluating the Restoration and Commercial Seed Growing Potential of Native Plants That Support Pollinators.” Restoration Ecology 34: e70202. 10.1111/rec.70202.

[ece374226-bib-0050] Urquhart‐Cronish, M. , C. S. Berg , D. Moxley , O. J. Rahn , L. Fishman , and A. L. Angert . 2025. “An Alpine Plant Shows no Decrease in Genetic Diversity Associated With Rapid Post‐Glacial Range Expansion.” American Journal of Botany 112: e70128. 10.1002/ajb2.70128. 41392371 PMC12712769

[ece374226-bib-0051] Van Hengstum, T. , D. A. P. Hooftman , H. C. M. Den Nijs , and P. H. Van Tienderen . 2012. “Does Insect Netting Affect the Containment of Airborne Pollen From (GM‐) Plants in Greenhouses?” Aerobiologia 28: 325–335. 10.1007/s10453-011-9237-8. 22798704 PMC3389241

[ece374226-bib-0052] Vickery, R. K. 1968. “Crossing Barriers in Mimulus.” Proceedings of the XII International Congress of Genetics, Tokyo, Japan 2:216–217.

[ece374226-bib-0053] Von Der Lippe, M. , and I. Kowarik . 2007. “Crop Seed Spillage Along Roads: A Factor of Uncertainty in the Containment of GMO.” Ecography 30: 483–490. 10.1111/j.2007.0906-7590.05072.x.

[ece374226-bib-0054] Vtipil, E. E. , and S. N. Sheth . 2020. “A Resurrection Study Reveals Limited Evolution of Phenology in Response to Recent Climate Change Across the Geographic Range of the Scarlet Monkeyflower.” Ecology and Evolution 10: 14165–14177. 10.1002/ece3.7011. 33391707 PMC7771151

[ece374226-bib-0055] Waits, J. , N. Larson , M. C. Moazed , A. Regan , M. Strebler , and L. Flores‐Rentería . 2025. “Phenological Variation and Evolution Across Space and Time in the Scarlet Monkeyflower ( *Erythranthe cardinalis* ).” Ecology and Evolution 15, no. 12: e72752. 10.1002/ece3.72752. 41426623 PMC12717473

[ece374226-bib-0056] Wang, L. , P. Haccou , and B.‐R. Lu . 2016. “High‐Resolution Gene Flow Model for Assessing Environmental Impacts of Transgene Escape Based on Biological Parameters and Wind Speed (S‐Q Huang, ed.).” PLoS One 11: e0149563. 10.1371/journal.pone.0149563. 26959240 PMC4784949

[ece374226-bib-0057] Wenzell, K. E. , M. Neequaye , P. Paajanen , et al. 2023. “Within‐Species Floral Evolution Reveals Convergence in Adaptive Walks During Incipient Pollinator Shift.” *bioRxiv*. 10.1101/2023.10.29.564637. PMC11923230 40108138

[ece374226-bib-0058] Whelan, R. J. , D. G. Roberts , P. R. England , and D. J. Ayre . 2006. “The Potential for Genetic Contamination vs. Augmentation by Native Plants in Urban Gardens.” Biological Conservation 128: 493–500. 10.1016/j.biocon.2005.10.016.

[ece374226-bib-0059] Wilbert, S. M. , D. W. Schemske , and H. D. Bradshaw . 1997. “Floral Anthocyanins From Two Monkeyflower Species With Different Pollinators.” Biochemical Systematics and Ecology 25, no. 5: 437–443. 10.1016/s0305-1978(97)00027-6.

[ece374226-bib-0060] Wilborn‐Pilotte, O. , E. Cook , K. Kutella , S. N. Sheth , and J. Diez . 2025. “Evolution of Floral Traits and Mating Systems Under Drought: A Range‐Wide Study of *Mimulus cardinalis* .” AoB Plants 17, no. 6: plaf062. 10.1093/aobpla/plaf062. 41323022 PMC12662168

[ece374226-bib-0061] Wooliver, R. , S. B. Tittes , and S. N. Sheth . 2020. “A Resurrection Study Reveals Limited Evolution of Thermal Performance in Response to Recent Climate Change Across the Geographic Range of the Scarlet Monkeyflower.” Evolution 74: 1699–1710. 10.1111/evo.14041. 32537737

[ece374226-bib-0062] Zhang, L. , L. Liu , Z. Fang , et al. 2023. “Fitness Changes in Wild Soybean Caused by Gene Flow From Genetically Modified Soybean.” BMC Plant Biology 23: 424. 10.1186/s12870-023-04398-2. 37710180 PMC10500775

